# Osteochondroma of the Scapula with Accessory Nerve (XI) Compression

**DOI:** 10.1155/2018/7018109

**Published:** 2018-01-31

**Authors:** Philippe Beauchamp-Chalifour, Stéphane Pelet

**Affiliations:** ^1^Centre de recherche FRQS du CHU de Québec, Hôpital Enfant-Jésus, 1401 18ème Rue, Ville de Québec, QC, Canada G1J 1Z4; ^2^Department of Orthopedic Surgery, CHU de Québec, Hôpital Enfant-Jésus, 1401 18ème Rue, Ville de Québec, QC, Canada G1J 1Z4

## Abstract

Osteochondroma is the most common benign bone tumor and is characterized as a cartilage-capped bony stalk. This lesion usually develops from the growth plate of long bones. Most osteochondromas are asymptomatic. Neurovascular compressions or cosmetic issues can occur in specific locations. Malignant transformation is extremely rare, and MRI can help evaluate these lesions. Symptomatic mass and malignancy features are the main surgical indications. Uncommonly, an osteochondroma can develop from flat bones. We present the case of a 25-year-old patient with a right scapula osteochondroma causing an accessory nerve compression. The mass was surgically removed, and the diagnosis was confirmed. The patient fully recovered at the latest 3-year follow-up visit.

## 1. Introduction

Osteochondromas account for 35 to 46% of benign bone tumors [1]. This tumor is characterized as a cartilage-capped osseous stalk with a bone marrow cavity in continuity to the underlying bone. This lesion usually develops from the growth plate of long bones in the first two decades of life, when the cartilage solidifies into the bone. Solitary osteochondromas are by far the most common presentation, and numerous lesions in the same patient are observed in the multiple hereditary osteochondromatosis.

Osteochondroma is generally asymptomatic and discovered during diagnostic radiological exams ordered for other purposes (i.e., search for fractures after a trauma). Two different morphological types are described: sessile, with an increased risk for malignant transformation, and pedunculated. The risk for malignant transformation into chondrosarcoma is thought to be less than 1%. Osteosarcoma transformation is very uncommon.

Depending on its location, an osteochondroma can sometimes cause neurovascular compression, cosmetic issue, and/or pain. This often leads to surgical removal with histopathologic analysis to confirm the diagnosis. Symptom disappearance with fully recovered function is generally expected [1–5].

Uncommonly, an osteochondroma can develop from flat bones. We present the case of a 25-year-old patient with a right scapula osteochondroma causing an accessory nerve (XI) compression. The mass was surgically removed through a posterior incision, and the diagnosis was confirmed. The patient fully recovered at the latest 3-year follow-up visit.

## 2. Case Report

A 25-year-old male presented at our clinics for a right shoulder pain related to a dorsal scapular mass first observed 4 months earlier. The main symptom was a shoulder discomfort when lying on his back, sometimes compromising the sleep. The patient also complained of some weakness when using his right arm under the shoulder level. The medical history consisted in a unique kidney and past treatments for a nodular sclerosis-subtype Hodgkin lymphoma (chemotherapy and radiotherapy, last treatment twelve years ago and no recurrence).

**Figure 1:**
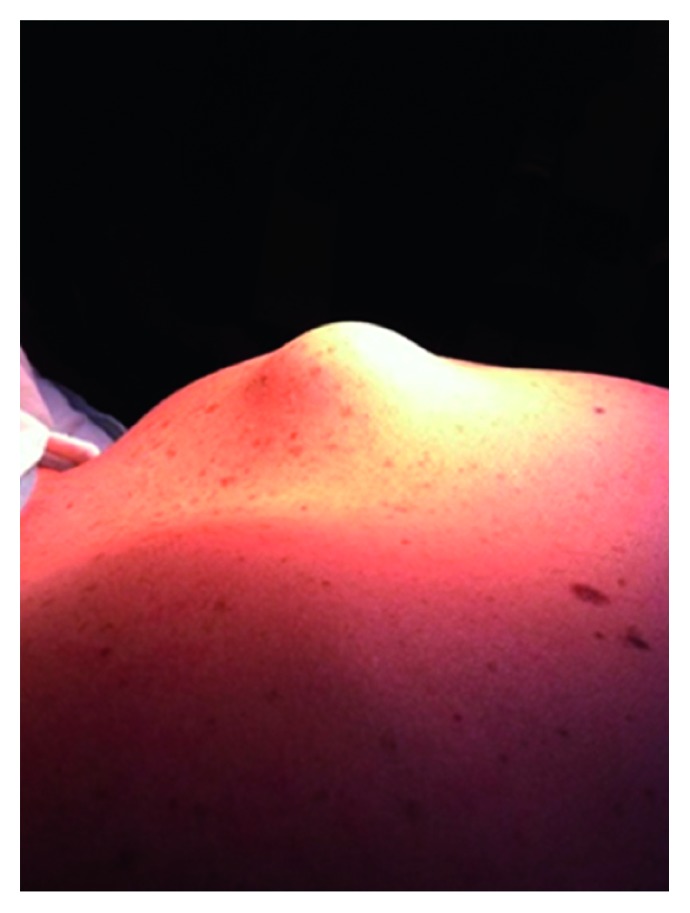
Nonmobile 4 cm mass at the posterior aspect of the scapula.

The physical examination revealed a nonmobile 3 cm T × 3 cm AP × 2 cm CC hard mass on the posterior superomedial angle of the right scapula, sensitive to palpation (Figure 1). Muscle trophicity was symmetrical. The right shoulder presented a full range of motion in all directions without scapulothoracic dyskinesis. Weakness and pain were observed when raising the right shoulder or during active abduction (at all levels).

**Figure 2:**
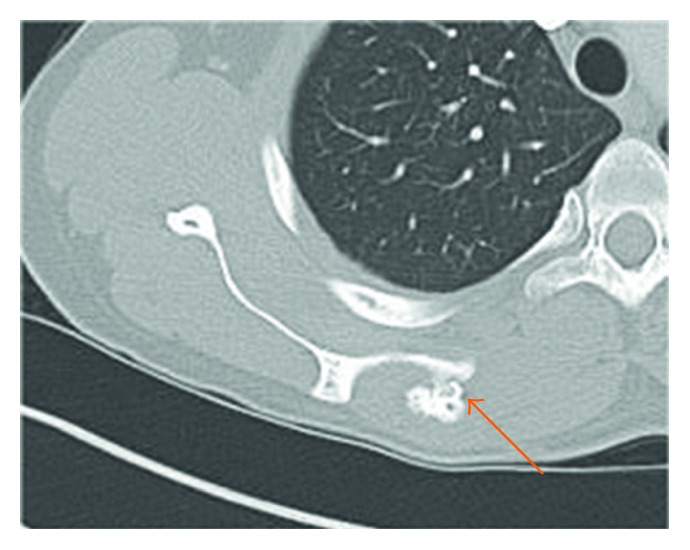
Thoracic CT scan axial view illustrating the solid mass measuring 2.6 cm T × 2.6 cm AP × 2.2 cm CC (orange arrow) at the superomedial aspect of the right scapula.

**Figure 3:**
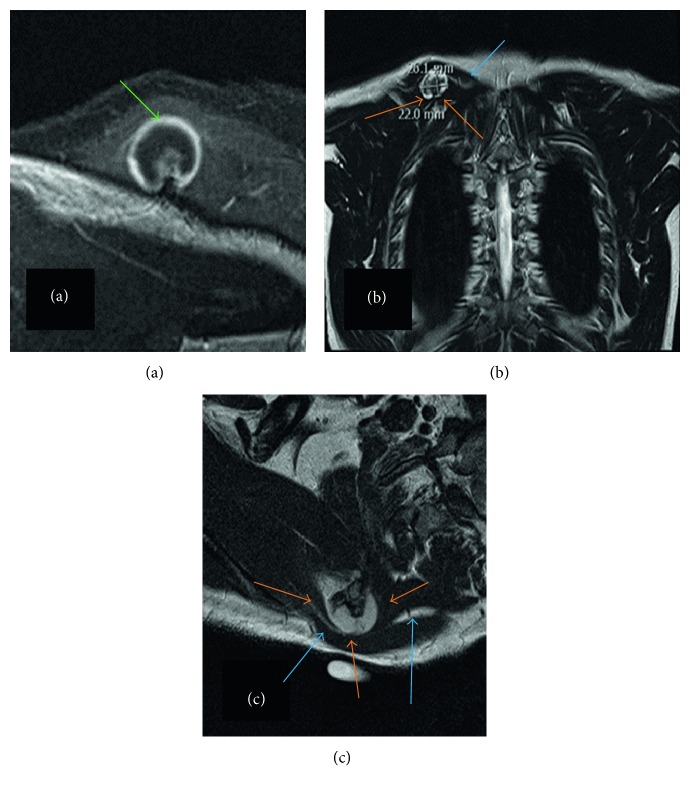
Thoracic MR imaging of the osteochondroma. T1 axial view (a) illustrating the benign 9 mm smooth cap (green arrow). T2 coronal (b) and sagittal (c) views showing the osteochondroma measuring 2.6 cm T × 2.6 cm AP × 2.2 cm CC (orange arrows) located on the right scapula and compressing the accessory nerve (blue arrows).

A right shoulder radiological series including AP, lateral, and Neer views was described normal and conducted additional investigations. A thoracic computed tomography was performed and described a 2.6 cm T × 2.6 cm AP × 2.2 cm CC solid bone mass at the posterior aspect of the superomedial angle of the scapula. The diagnosis of an osteochondroma without signs of malignancy was stated (Figure 2). No adenopathy or recurrence of the past Hodgkin lymphoma was observed. Retrospectively, the mass was apparent on the shoulder Neer view. A magnetic resonance imaging (MRI) including the right shoulder and scapula confirmed the benign character of the osteochondroma. The accessory nerve (XI) was compressed between the osteochondroma and the deep layer of the trapezius; no muscle atrophy was described (Figure 3).

**Figure 4:**
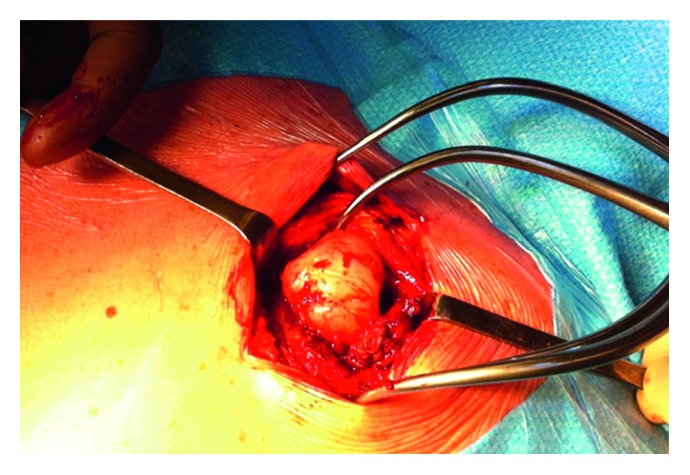
Surgical view of the osteochondroma. The lesion measured 3 cm T × 3 cm AP × 2 cm CC.

**Figure 5:**
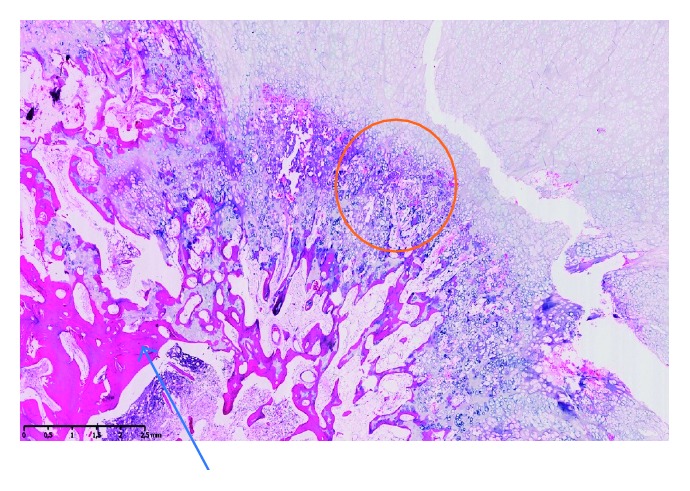
Histopathological section of the osteochondroma illustrating the bony trabeculae (blue arrow) and the cartilage component of the lesion (orange circle).

The osteochondroma was surgically removed under general anesthesia. The patient was prone, and a 10 cm incision was centered on the mass. The trapezius muscle was split at its superior part at the level of the scapula spine. The accessory nerve was visualized and protected in the deep layer of the trapezius muscle. The osteochondroma was exposed and resected from the supraspinatous fossa with an osteotome. The mass was perfectly smooth and measured 3 cm T × 3 cm AP × 2 cm CC (Figure 4). The integrity of the accessory nerve was checked before closure. The histopathologic analysis confirmed the diagnosis of a benign osteochondroma (Figure 5).

The shoulder was immobilized in a sling 10 days for wound care. Free mobilization was then granted. No physiotherapy was needed.

The patient was followed annually up to 3 years. He regained full shoulder range of motion and complete symmetric trapezius and rotator cuff strength. The patient has no clinical and radiological recurrence of the osteochondroma.

## 3. Discussion

Osteochondroma was initially thought to represent a misplaced epiphyseal cartilage plate herniated in a bone with a periosteal defect. Recent basic research on gene analytics considered these tumors as true primary neoplasms. Most cases represent solitary lesions. Multiple lesions are observed in the multiple hereditary osteochondromatosis. A familial link with germline mutations of EXT1, EXT2, and EXT3 was described in 90% of patients. EXT1 mutation (8q24) was even found in some cases with a solitary lesion.

In this case, we questioned a possible genetic link between the past diagnosis (unique kidney and Hodgkin lymphoma) and the osteochondroma. However, no association was found on the Online Mendelian Inheritance in Man (OMIM) database.

Secondary osteochondromas have been reported with autologous hematopoietic stem cell transplant, trauma on the growth plate, and irradiation. Osteochondroma is the most common benign radio-induced tumor. Dosage from 1.25 grays (Gy) up to 64.25 Gy was described for radio-induced osteochondroma, with a mean latent time of 8 years. In this case, the patient received chemotherapy and mantle field radiotherapy for his nodular sclerosis-subtype Hodgkin lymphoma. Even though mantle field irradiation is relatively precise, no specific shields are used to block the radiations outside the dedicated zone (scapula, lungs, and humeral head). The patient received 14 cycles of mantle field irradiation for a total absorbed dose of 21 Gy. Considering that the last irradiation dated twelve years ago and no lesion was observed on the last chest tomodensitometry (seven years ago), a link between the irradiation and the osteochondroma is less likely.

Flat bone is an uncommon site for solitary osteochondroma [1–12]. Most reported cases on the scapula tend to develop on the ventral side, causing snapping and pseudowinging. To our knowledge, this case represents the first osteochondroma arising from the posterior superomedial angle of the scapula. This specific location is propicious to affect the surrounding neurologic structures, mainly the accessory and suprascapular nerves. The patient demonstrated trapezius weakness, and the imaging illustrated the compressed accessory nerve between the osteochondroma and the deep layer of the trapezius. We did not ask for an electromyogram to assess the level of impairment of the accessory nerve as it would not have changed the need for surgical removal. Only the surgical approach was adapted to protect the nerve during the deep dissection. The patient recovered full strength at the latest follow-up, confirming full decompression of the accessory nerve.

Various tumors can affect the scapula. Osteochondroma is the most frequent benign tumor, and chondrosarcoma is the most frequent malignant one [13]. The location on the scapula can help for the differential diagnosis. The Musculoskeletal Tumor Society divided the scapula into two zones: the S1 zone includes the blade-spine portion of the scapula and the S2 zone the acromioglenoid complex. Although benign tumors are more likely to sit in S2 zone (including aggressive benign tumors like aneurysmal bone cyst and giant cell tumor), osteochondromas are often observed in S1 zone, a zone more prone to malignancies (chondrosarcoma, Ewing's sarcoma, multiple myeloma, and lymphoma). In addition to the location, the clinical presentation and imaging (mainly MRI) are very helpful to differentiate malignancies from benign tumors.

A rare entity called Nora lesion can mimic an osteochondroma. These lesions are outgrowth from the cortical surface characterized by the bone, cartilage, and fibrous tissue and affect mostly patients in their 20s [14]. The most common affected bones are phalanges and metacarpal and metatarsal bones [14]. Otherwise, osteochondromas are continuous to the cortical bone, and the histopathologic analysis is essential for the final diagnosis.

Osteochondromas are best illustrated with MRI, mainly the extension of the native medullary cavity into the tumor's marrow cavity. The cartilaginous cap is richer in water than the bone part, and the cartilage thickness can be easily measured: a thickness higher than 1.5 to 2 cm in adults and 3 cm in children is suspect for a malignant transformation into a low-grade osteosarcoma [15]. Other malignant features well detected on MRI are prominent myxoid changes, wide fibrous bands, lobulations, and enhancement after gadolinium injection. Furthermore, MRI is useful to analyze the tumor's impact on the local contiguous structures: reactive bursitis, nerve compression, vascular compression, and muscle denervation [15]. In this case, MRI was very helpful to describe the benign character of the lesion and the accessory nerve compression.

To our knowledge, this case is the first case illustrating a posterior scapular osteochondroma with accessory nerve compression. This demonstrates the need for careful clinical and radiological evaluation of these tumors, mostly to identify the possible compression of the surrounding neurovascular structures.
